# Heart Rate Variability—An Index of the Efficacy of Complementary Therapies in Irritable Bowel Syndrome: A Systematic Review

**DOI:** 10.3390/nu14163447

**Published:** 2022-08-22

**Authors:** Magdalena Mróz, Marcin Czub, Anna Brytek-Matera

**Affiliations:** Institute of Psychology, University of Wrocław, 50-527 Wrocław, Poland

**Keywords:** irritable bowel syndrome, heart rate variability, complementary therapies, mind-body therapies

## Abstract

Irritable bowel syndrome (IBS), as a functional and psychosomatic disease, reduces the quality of life and increases the risk of developing mental disorders. Deregulation of the autonomic nervous system (ANS) is one of the main causes of the disease. The objective of the present study was to identify the studies in which measurements of heart rate variability (HRV) were performed before and after therapeutic intervention, and to evaluate the effectiveness of IBS therapy in terms of a reduction of IBS symptoms and changes in autonomic tone. A systematic review of the literature was carried out in accordance with PRISMA standards. Six databases were searched for articles published before 2022: PubMed^®^, MEDLINE^®^, EBSCO, Cochrane, Scopus, and Web of Science. Inclusion criteria were experimental design, diagnosis of IBS (medical and/or diagnosis in accordance with the Rome Criteria), non-pharmacological intervention, and HRV measurement before and after the intervention. The quality of the studies was assessed by JBI Critical appraisal. In total, 455 studies were identified, of which, sixwere included in the review. Expected changes in HRV (increase in parasympathetic activity) were observed in four of the six studies (interventions studied: ear acupressure, transcutaneous auricular vagusnerve stimulation, cognitive behavioral therapy with relaxation elements, yoga). In the same studies, therapeutic interventions significantly reduced the symptoms of IBS. The present review indicated that interventions under investigation improve the efficiency of the ANS and reduce the symptoms of IBS. It is advisable to include HRV measurements as a measure of the effectiveness of interventions in IBS therapy, and to assess autonomic changes as a moderator of the effectiveness of IBS therapy.

## 1. Introduction

Irritable bowel syndrome (IBS) is a functional gastroenterological disorder. It is characterized by recurrent visceral pain (on average, one day a week for the last 3 months), along with changes in bowel movements (constipation and/or diarrhea) [1]. The global prevalence of IBS is 13.8% among women and 9.4% among men [2]. The chronic course [3] and nuisance of symptoms reduce the quality of life and performance at work [4], and intensify the symptoms of mood disorders, anxiety, or somatic disorders [5,6].

**Figure 1:**
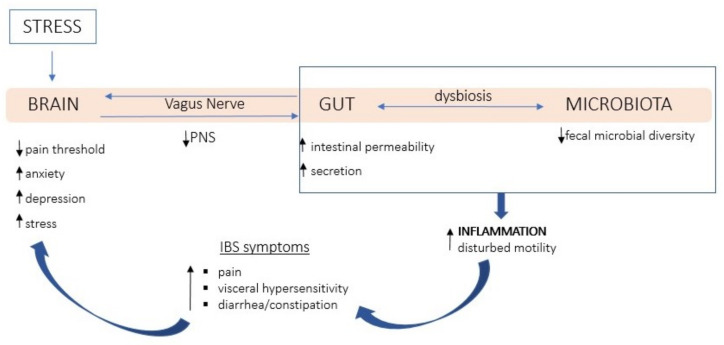
Schema the relationship between disturbed autonomic balance and IBS symptoms. Note: Permanent stress and negative emotions inhibit the vagus nerve function, which manifests itself in hypoactivity of the PNS. Under the stress, the homeostasis of the intestinal epithelium is disturbed, and the microbiota composition is modified, e.g., reduction of fecal microbial diversity, which may lead to dysbiosis. Impaired secretory and barrier functions of the intestinal mucosa are characterized by increased intestinal permeability and secretion, which contribute to the development of intestinal inflammation and disturbances in intestinal motility. Inflammation causes pain, problems with defecation (diarrhea/constipation), and the visceral hypersensitivity increases. These symptoms affect neurophysiological changes (including lowering the pain threshold) and mental changes (increased anxiety, depression) in the brain, which secondarily generates stress and reduces the anti-inflammatory efficiency of the vagus nerve. This simplified elaboration is based on Bonaz et al., 2018 [11] and Drossman, 2016 [1].

The complex etiology of IBS is mainly described as microbiota–gut–brain interaction disorders [1]. Moreover, the intestinal symptoms get worse under stress [7]. From the perspective of this review, the key feature of IBS is the disturbed autonomic balance, which is characterized by a reduced vagal tone and reduced parasympathetic nervous system activity [8,9,10]. The relationship between disturbed autonomic balance and IBS symptoms is presented in Figure 1. The parasympathetic nervous system is mainly based on bi-directional communication running through the vagus nerve. Under the influence of stress, the activity of the vagus nerve decreases (decrease of parasympathetic activity), which causes dysbiosis and increases the secretion and permeability of the intestines. As indicated by Bonaz et al. [11], it increases inflammation in the intestines. Afferent nociceptors become more sensitive, which increases the sensation of visceral pain in IBS [12]. In IBS, visceral nociception is also intensified due to functional and structural changes in the brain, immune, and neuroendocrine pathways [13].

Symptomatic treatment (also in pharmacotherapy) dominates in IBS therapy, which aims to alleviate symptoms. The non-pharmacological methods of therapy include cognitive–behavioral therapy (CBT) [14], intestinal-oriented hypnosis [15], mind–body therapies (MBT) [16], mindfulness therapies (e.g., MBSR) [17], herbal medicine [18], and probiotics [19]. A change in diet and lifestyle is also recommended [20,21]. After reviewing the literature, Black and Ford [20] propose that the order of applying the therapy should begin with a lifestyle change, through diet, and, ultimately, pharmacotherapy. Nelkowska [21] proposes to combine conventional medicine with alternative therapies. As each subsequent ineffective form of therapy extends the time of suffering and exposes to the risk of secondary mental disorders or the development of other functional disorders, it is advisable to look for tools that will enable verification of the effectiveness of therapies in terms of their impact on the cause of disorders, i.e., deregulation of the autonomic balance.

Commonly used measures of the effectiveness of IBS therapy are observational forms (diaries of abdominal symptoms) and questionnaires assessing the occurrence and severity of IBS symptoms (Global Improvement Scale, IBS-Severity Scoring System, IBS-Quality of Life, pain scales); the level of quality of life; and the level of stress, mood (anxiety, depression), and visceral hypersensitivity. These measures are self-descriptive, mostly retrospective. Physiological and medical measures include biomarkers from blood (markers of inflammation, concentration of neurotransmitters, e.g., serotonin), which are additionally carried out [22,23]. On the other hand, HRV recordings are used as a measure of autonomic changes.

Heart rate variability (HRV) is an indirect measure of autonomic functions—among others, it is an indicator of parasympathetic nervous system activity [24]. HRV reflects the fluctuations in the time intervals between successive heartbeats that result from the interaction of the body’s regulatory systems. Therefore, HRV reflects the body’s ability to adapt to psychological and environmental challenges [25]. HRV measurement is seemingly easy, but requires appropriate methodological conditions and researchers’ awareness in terms of analysis and the interpretation of the obtained results [26]. Dedicated HRV parameters that present changes in parasympathetic ANS activity, including, in particular, changes in the vagal tone, are: RMSSD (root mean square of successive differences between normal heartbeats), pNN50 (percentage of adjacent NN intervals that differ from each other by more than 50 ms), and HF (high-frequency power) [26]. On one hand, an assessment of the effectiveness of irritable bowel syndrome therapy through HRV may supplement subjective measurements. On the other hand, it broadens the possibilities of interpreting the observed changes. It results from the relationship of the parasympathetic nervous system with health; self-regulation mechanisms; and cognitive, affective, and social functioning [27,28].

In IBS studies, autonomic function measurements are performed to assess differences in ANS performance between IBS patients and the healthy ones [29]; and IBS patients and participants suffering from other disorders and diseases [30,31]. Another goal of HRV measurements is to differentiate IBS subtypes (C/D/M, IBS with constipation-predominant symptoms, IBS with diarrhea-predominant symptoms, IBS with mixed bowel habits) [32]. Changes in the functioning of the ANS in connection with a meal [33] or the predictive possibilities of the effectiveness of therapy based on resting HRV are also assessed [34].

The development of the possibility of using HRV measurements in the IBS characteristics has been presented in the previous reviews. In the first literature review, Park [35] described the possibilities of using HRV measurements in IBS research. Mazurak et al. [32] indicated that HRV values differ depending on the IBS subtype. Liu et al. [36] reported on differences in autonomic tension between healthy and IBS patients, which empirically supported assumptions about impaired parasympathetic nervous system efficiency and impaired sympathetic-vagal balance in IBS. The most recent meta-analysis [29] focused on the analysis of the differences in resting HRV (HF) between healthy and IBS patients. Again, they showed that the parasympathetic nervous system is less active in individuals with IBS compared to healthy ones. However, there has not yet been a review that focuses on the possibilities of using HRV measures in assessing the effectiveness of therapeutic interventions in IBS.

The evaluation and selection of IBS therapies in terms of their impact on the autonomic nervous system (ANS) tone, and especially the vagus nerve, may change the treatment of IBS from symptomatic to directed at the main pathomechanism (disorders of microbiota–gut–brain interaction) [1]. Planning therapies that will increase vagal tone (increasing parasympathetic activity of ANS) will reduce inflammation in the intestines (e.g., by reducing secretion and intestinal permeability) [11]. As a result, it will improve the quality of physical and mental life, limit the development of mental disorders, and increase productivity at work. From an economic perspective, a faster selection of effective therapy will reduce the costs of treatment. Better productivity at work will reduce the economic and social costs of the illness. Due to the high clinical and social importance of this topic, the present review sets out three objectives: (1) the identification of studies in which HRV measurements were performed before and after therapeutic interventions; (2) the evaluation of the effectiveness of IBS therapy in terms of a reduction of IBS symptoms (diagnostic and co-occurring) and changes in autonomic tone; (3) the assessment of the effectiveness of using HRV measures as an indicator of the effectiveness of IBS therapy.

## 2. Methods

### 2.1. Search Strategy

The literature for the review was found in electronic databases in English: PubMed^®^, MEDLINE^®^, EBSCO, Cochrane, Scopus, and Web of Science. The search terms were: Irritable Bowel Syndrome OR IBS AND Heart Rate Variability OR HRV. While reviewing databases, in the case of three of them (Cochrane, Scopus, Web of Science), the TIAB filter was used; the remaining databases were searched comprehensively. The material search was completed in February 2022. All studies were reviewed by the first author (M.M.), and all included studies were additionally reviewed by the second author (M.C.).

### 2.2. Search Strategy

**Figure 2:**
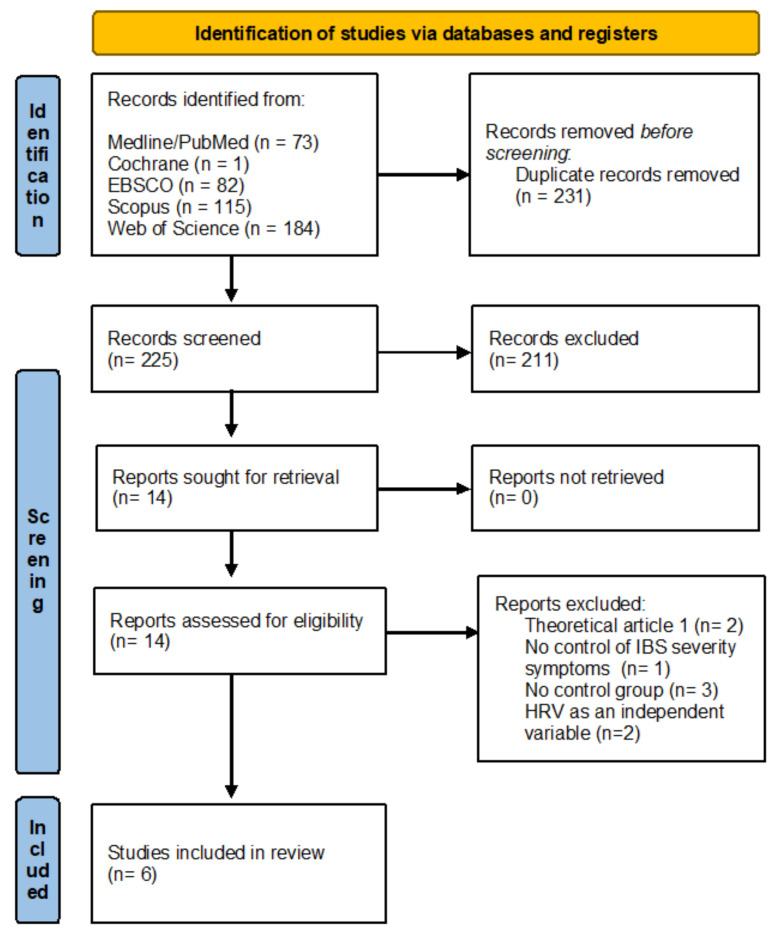
Flow diagram for systematic review.

The criteria for the inclusion of studies in the review were: the diagnosis of IBS based on a medical examination and/or in accordance with the Rome Criteria. In addition, the studies which had an experimental design with the control group were included, where HRV was measured twice (before and after the intervention). The therapeutic intervention had to be non-pharmacological—body–mind or psychophysical therapy. Figure 2 shows the process of qualification of studies for the review, and details the reasons for exclusion.

### 2.3. Primary Outcomes

The primary measurements consisted of assessments of the severity of symptoms of irritable bowel syndrome (e.g., IBS—Severity Scoring System [37], or analogous questionnaires indicating the severity of disease symptoms). As for autonomic function measurements, indicators of the heart rate variability describing changes in parasympathetic activity in the vagus nerve tone were sought. In the HRV time domain, these were the RMMSD and pNN50 indicators, and in the frequency domain—the HF indicator [25]. The description of the results included indicators expressed in standard values, normalized, or converted by natural logarithm.

### 2.4. Secondary Outcomes

Secondary measurements concerned the severity of pain and the co-occurring symptoms of IBS: the level of experienced stress, anxiety, and depression. Psychological factors are not included in the formal criteria for the diagnosis of IBS. However, they are related to the severity of symptoms and the decision whether to use medical, psychological, or dietary help [1]. Abdominal pain is the most disturbing symptom of IBS for women [38], and stress is an important risk factor for the development of IBS and the severity of the experienced pain [7]. The symptoms of IBS are associated with the severity of the symptoms of mental disorders, and most often occur with anxiety and mood disorders [5].

### 2.5. Synthesis of Results

We used PRISMA guidelines in the evaluation of the results [39]. Due to the diversity of the reported data, a qualitative synthesis of results was developed, divided into five categories: (1) general characteristics of the research; (2) characteristics of the respondents and experimental interventions; (3) methodology of HRV measurement; (4) comparison of the size of effects for primary and secondary variables; (5) quality of the reported research.

### 2.6. Evaluation of the Quality of Tests

Two scales were used to assess the risk of study bias, which independently evaluated the quality of randomized controlled trials (RCTs) and non-randomized experimental trials (non-RCTs). The JBI Critical Appraisal Checklist for Quasi-Experimental Studies (non-randomized experimental studies) consists of nine questions, and the JBI Critical appraisal checklist for randomized controlled trials consists of 13 questions. Depending on the scale, the quality assessment of the study is in the range of 0–9 or 0–13 points, where 0 means a high risk of bias in the study, and 9 or 13—a low risk of bias in the study. The selected scales are dedicated to the assessment of research that focuses on the observation of the effectiveness of the experimental intervention [40].

## 3. Results

In total, 455 studies were identified. No additional studies were identified based on the source literature. The selection process is presented in Figure 2. Finally, 12 studies measured HRV before and after therapeutic interventions, six of which were included in this review. Other studies did not meet the inclusion criteria due to: a different type of intervention (herbal medicine [41] and pharmacotherapy [42]), the lack of a passive control group [34,43,44], and the lack of an assessment of changes in the severity of IBS symptoms [45].

### 3.1. Characteristics of the Studies

A brief description of all studies included in the review is provided in Table 1, Table 2, Table 3 and Table 4. Table 1 summarizes the characteristics of each study.

Table 2 expands on the description of the applied intervention for the experimental and control groups.

**Table 2:** Characteristics of the experimental interventions used to treat IBS symptoms.

Study	Intervention (E/C)	Duration/Frequency/ Duration of One Session	Description
Jang et al. [46]	CBT/general information about IBS	E: 8 weeks/once a week/ 80 min (include 20 min of relaxation training); C: 1st week/only once/50 min	E: Group session of CBT (4–6 participants) with 60 min of thematic training and 20 min of relaxation training. C: One session with 50 min of general information about IBS (during the first week). Similarly to the experimental group—an interview on IBS symptoms in groups of 4–6 people (four times: at baseline; and at 8, 16, and 24 weeks).
Jurek et al. [47]	Slow deep breathing/normal activities	E: 4 weeks/5 times a week/20-min; C: normal activities	E: Self-directed Slow Deep Breathing with 20-min video. At least 4 times a week. C: maintenance regular activity.
Kavuri, Selvan, Malamud et al. [48]	Yoga/Combination/ Wait-List groups ^a^	Y/CB—Yoga RYM: 12 weeks/ 3 times a week/60 min; WL-C -walking: 12 weeks/once a day/60 min	E: Each yoga session started with simple breathing practices, loosening practices, and simple postures with relaxation in between. The session ended with regulated breathing and meditation. C: maintenance of their regular activities; suggestion to walk for 60 min three times a week during their waiting period.
Park and Cha [49]	KHA/sham-KHA	4 weeks/twice a week/25 min	E: 16 KHA reflection spots on both hands were stimulated. The needles were inserted at less than 1 mm depth. C: 16 spots that were unrelated to the crucial energy spots were inserted by the needles. Each reflection spot wasstimulated for 25 min in both groups.
Go and Park [50]	Auricular Acupressure/ no treatment	4 weeks/5 days a week/5 times a day	E: Semen sinapis albae seeds were used to acupressure four auricular points: endocrine, large intestine, lung, and Shenmen. Stickers remained in place for 5 days, and sticker-attached areas were pressed 5 times a day. Acupressure stickers were applied weekly for 4 weeks with a 2-day break time between each treatment. C: No treatment.
Shi et al. [51]	taVNS/ sham-taVNS	4 weeks/twice a day/30 min	E: “The taVNS treatment was performed at auricular cymba concha. One pair of electrodes was placed at bilateral auricular concha, via which trains of pulses were delivered from a watch-size digital stimulator” [51] p. 12. C: Sham-taVNS was performed with the same parameters as taVNS. Electrical stimulation was performed at sham point at the elbow area.

C = control group; CBT = cognitive behavioral therapy; E = experimental group; KHA = Korean hand acupuncture; RYM = remedial yoga module; taVNS = transcutaneous auricular vagusnerve stimulation; ^a^ Two experimental groups: (Y) yoga with limited conventional treatment, and (CB) yoga with conventional treatment; one control group: wait-list.

### 3.2. Characteristics of HRV Measurements

Table 3 refers to the HRV measurement methodology. In the analyzed studies, short-term HRV measurement was most often performed in the range of 5 min to 30 min, in a seated (three studies) or supine (two studies) position. Measurement details were not reported in the study by Jurek et al. [47]. None of the studies reported at what time of day the measurements were made (and whether the time was constant for repeated measurements in the same person). The bands of the analyzed frequencies were indicated in two studies. HRV was measured using an ECG apparatus (electrodes) (three studies), a finger measurement (two studies), or a chest pulse sensor (one study). HRV parameters were generated using dedicated computer software. The reported HRV parameters differed between studies. Frequency domain parameters (HF, LF—normalized/logged equivalents, and LF/HF coefficient) were most often reported. Studies by Go and Park [50], and Park and Cha [49] additionally indicated time domain parameters (SDRR). A study by Jurek et al. [47] reported complex parameters generated on the basis of several basic HRV parameters, e.g., PNS index (parasympathetic nervous system activity compared to normal resting values [52]).

**Table 3:** Methodology of HRV measurement.

Study	Position and Length of Recordings	Time of HRV Recording	Frequency Ranges (Hz)	HRV Hardware	HRV Software	HRV Indicators
Jang et al. [46]	seated, 10 min	unclear	HF: 0.15–0.4 LF: 0.04–0.15	QECG-3 monitoring system (Laxtha Inc., Daejeon, Korea)	TeleScan Ver.2.8; Laxtha Inc.	HF, LF/HF
Jurek et al. [47]	unclear	unclear	unclear	Polar heart rate monitor (Kempele, Finland)	Elite HRV app and Kubios software (Finland)	HF, LF/HF, PNS index, SNS index
Kavuri, Selvan, Malamud et al. [48]	lying, 5 min	unclear	unclear	ECG and respiration—Biopac MP 45 Data Acquisition System (BIOPAC, CA, USA)	Kubios (version 2.2, Finland)	HF, LF, LF/HF
Park & Cha [49]	seated, 5 min	unclear	unclear	SA-3000P (Medicore Co. Ltd., Seoul, Korea)		SDRR, PSI, TP, VLF, LF, HF, LF-Norm, HF-Norm, LF/HF
Go & Park [50]	seated, twice ^a^ for 5 min	unclear	unclear	SA-3000P (Medicore Co. Ltd., Seoul, Korea)		SDRR, PSI, TP, LF-Norm, HF-Norm, LF/HF
Shi et al. [51]	lying, 30 min	unclear	HF: 0.15–0.50 LF: 0.04–0.15	ECG-01A (Ningbo Maida Medical Device Inc., Ningbo, China)	unclear	LF-Norm, HF-Norm

ECG = electrocardiogram; HF = high frequency; HF-norm = normalized high frequency (HF/LF + HF); LF = low frequency; LF/HF = low frequency/high frequency ratio; LF-norm = normalized low frequency (LF/LF + HF); PNS index = parasympathetic nervous system index; PSI = physical stress index; SDRR = standard deviation of RR intervals; SNS index = sympathetic nervous system index; TP = total power; VLF = very low frequency; ^a^ average for analysis.

### 3.3. Evaluation of the Effectiveness of the Intervention

Table 4 presents the results and conclusions from the analyzed studies. In the analyzed studies, expected changes in HRV (increase in parasympathetic activity assessed with: HF, HF-Norm, LF/HF) were observed in four of the six studies [46,48,50,51]. In the Park and Cha study [49], there were no comparisons of post-tests between groups, and tests for intra-group differences did not show the impact of interventions on changes in ANS.

The use of therapeutic interventions significantly affected the reduction of IBS symptoms coexisting with an increase in parasympathetic activity of ANS in four of the six analyzed studies [46,48,50,51].

The severity of pain was measured in three of six studies [49,50,51], of which, two demonstrated a significant decrease in pain in the intervention group [50,51]. In the studies [49,50], the pain was assessed with three aspects of BSSS (frequency, discomfort, and the feeling of not fully functioning). Shi et al. [51] assessed changes in pain intensity on the VAS scale.

Five studies measured the severity of anxiety and depression. A significant reduction in anxiety and depression was observed after three interventions [46,48,51]. The measurement of anxiety and depression in the Go and Park [50], and Park and Cha [49] studies was performed with the SCL-90R-K subscale. The authors note that the scale was aimed at detecting psychopathology (which was not confirmed in the study participants). The scale may have been insufficiently sensitive to changes in symptom severity.

Three studies measured the stress level [46,49,50]. In all of them, a reduction in stress was observed in the intervention group as compared to the control group. Go and Park [50] showed significant differences based on the test for independent groups, where the changes in the stress level before and after the intervention (difference in results of post-test minus pre-test; t = −5.29, *p* < 0.001) were compared. Jang et al. [46] indicated only the significance (*p* < 0.001) of the difference in the average results of the post-test between the groups. Park and Cha [49], however, did not report results for intergroup differences in post-tests. However, we calculated the effect size using available information, and the results indicated an average effect (d = −0.51); the intervention group experienced less stress than the control group.

Two studies looked at the relationship between changes in HRV and the severity of IBS symptoms. In the study by Shi et al. [51], it was shown that both HRV (HF-norm) (r = −0.347; *p* = 0.025) and quality of life (IBS-QoL) (r = −0.422; *p* = 0.005) are negatively correlated with the intensity of pain. This means that the severity of pain decreased with the increase in parasympathetic activity and the perceived quality of life. This may mean that an increase in parasympathetic activity has reduced pain sensation, which, in turn, has improved the quality of life.

On the other hand, the balance of the functioning of the ANS (LF/HF) in the study by Jang et al. [46] was significantly positively correlated with changes in IBS symptoms, anxiety, depression, and stress. The increase in parasympathetic activity (HF) was negatively correlated with changes in IBS symptoms, anxiety, depression, and stress. These changes were observed in the follow-up which took place 12 weeks after the end of the intervention. Immediately after the intervention, a positive correlation between LF/HF and depression was observed (r = 0.44; *p* < 0.01). The relationship between changes in HRV and the severity of IBS symptoms has not been investigated in the Go and Park studies [50]. However, the observed strong magnitude of effects (Table 4) of changes in the severity of IBS symptoms and HRV indicates a similar direction of dependence as in the studies described above.

**Table 4:** Results and conclusions of the studies included in the review. Comparison of the size of effects for primary and secondary variables.

Primary and Secondary Variables	Methods for Assessing Variables	Effect Sizes ^a^	Significance Level	Conclusions
**Shi et al.** [51]				
IBS symptoms	IBS-SSS	d = 1.30	*p* = 0.001	taVNS improved HRV parameters—increased the vagal activity (HF-norm). taVNS reduced IBS symptoms, pain, anxiety, and depression, and improved quality of life.
HRV	HF-norm	d = −0.66	*p* = 0.04	
	LF-norm	n/d	n/d	
Pain	VAS	d = 1.17	*p* = 0.001	
Anxiety	SAS	d = 1.24	*p* < 0.001	
Depression	SDS	d = 0.84	*p* = 0.011	
Stress	not measured			
**Go and Park** [50]				
IBS symptoms	BSSS-AD-F	d = 0.81	n/d	Auricular acupressure effectively reduced IBS symptoms. The severity of loose stools, diarrhea, abdominal pain, and abdominal discomfort were lower. In the experimental group, HRV parameters significantly improved, indicating increased parasympathetic activity (increase in HFNorm), increased resistance to stress (increase in SDRR), and decreased LH/HF balance. The level of experienced stress in the experimental group decreased significantly.
	BSSS-AD-DS.	d = 1.16	n/d	
	BSSS-AD-DB	d = 1.52	n/d	
HRV	HF-norm	d = −1.1	n/d	
	LF-norm	d = 0.88	n/d	
	LF/HF	d = 0.88	n/d	
	PSI	d = 0.29	n/d	
	SDRR	d = −0.59	n/d	
Pain	BSSS-AP-F	d = 0.42	n/d	
	BSSS-AP-DS.	d = −0.70	n/d	
	BSSS-AP-DB	d = 0.97	n/d	
Anxiety	SCL-90R-K-A	d = 0.24	n/d	
Depression	SCL-90R-K-D	d = 0.13	n/d	
Stress	PSS	d = 1.07	n/d	
**Park and Cha** [49]				
IBS symptoms	BSSS-AD-F	d = 0	n/d	Some of the symptoms of IBS have improved—especially those related to abdominal pain: frequency of loose stools and abdominal pain, reduction of anxiety and perceived disability caused by abdominal pain, flatulence, and discomfort in the abdominal cavity. KHA was not effective in reducing stress and promoting mental health. There was no change in HRV.
	BSSS-AD-DS.	d = −0.32	n/d	
	BSSS-AD-DB	d = −0.24	n/d	
HRV	HF-norm	d = 0.18	n/d	
	HF	d = −0.39	n/d	
	LF-norm	d = −0.18	n/d	
	LF	d = −0.24	n/d	
	LF/HF	d = −0.20	n/d	
	PSI	d = 0.48	n/d	
	SDRR	d = −0.42	n/d	
Pain	BSSS-AP-F	d = 0.19	n/d	
	BSSS-AP-DS.	d = 0.15	n/d	
	BSSS-AP-DB	d = −0.32	n/d	
Anxiety	SCL-90R-K-A	d = −0.11	n/d	
Depression	SCL-90R-K-D	d = −0.05	n/d	
Stress	GARS	d = −0.51	n/d	
**Jurek et al.** [47]				
IBS symptoms	IBS-SSS	d = −0.19	n/d	There were no changes in the functioning of the autonomic system (no significant differences in HRV). The severity of IBS symptoms has not changed.
HRV	HF	d = 0	n/d	
	LF/HF	d = 0.16 eta2 = 0.47	n/d	
	PNS index	d = 0.80	n/d	
	SNS index	d = 0.18	n/d	
Pain	not measured			
Anxiety				
Depression				
Stress				
**Jang et al.** [46]				
IBS symptoms	GSRS-IBS	n/d	*p* < 0.001	Significant changes in the functioning of ANS were observed—CBT resulted in a significant increase in HF and a significant decrease in the LF/HF ratio. These changes coexisted with significant reductions in IBS symptoms, anxiety, depression, and stress. Differences in HF and the LF/HF ratio were significantly associated with changes in symptoms of IBS, anxiety, depression, and stress.
HRV	HF	n/d	*p* = 0.017	
	LF/HF	n/d	*p* = 0.003	
Pain	not measured			
Anxiety	HADS-A	n/d	*p* < 0.001	
Depression	HADS-D	n/d	*p* < 0.001	
Stress	GARS	n/d	*p* < 0.001	
**Kavuri**, Selvan, Malamud **et al.** [48]				
IBS symptoms	IBS-SSS	Y vs. WL: d = 4.03	*p* < 0.001	The remedial yoga module (RYM) reduces symptoms of IBS, anxiety, and depression (in both groups: (Y), yoga with limited conventional treatment; and (CB), combination—yoga with conventional treatment). Only in the combination group (yoga with conventional treatment) were there significantly favourable changes in HRV parameters (indicating increased parasympathetic activity).
		CB vs. WL: d = 3.12	*p* < 0.001	
		Y vs. CB d = 0.52	*p* > 0.05	
HRV	HF	n/d	CB vs. WL: *p* < 0.01	
	LF	n/d	CB vs. WL: *p* < 0.05	
	LF/HF	n/d	CB vs. WL: *p* < 0.01	
Pain	not measured			
Anxiety	HADS-A	n/d	Y vs. WL ***	
		n/d	CB vs. WL ***	
		n/d	Y vs. CB	
Depression	HADS-D	n/d	Y vs. WL ***	
		n/d	CB vs. WL ***	
		n/d	Y vs. CB	
Stress	not measured			

IBS-SSS, IBS Symptom Severity Scale; BSSS-AD, Bowel Symptom Severity Scale; AD, abdominal discomfort: F = frequency, DS = distress, DB = disability; AP = abdominal pain; GARS, Global Assessment of Recent Stress; GSRS-IBS, GI Symptom Rating Scale; HADS, Hospital Anxiety and Depression Scale; HF, high frequency; HF-norm, normalized high frequency (HF/LF + HF); HRV = heart rate variability; KHA, Korean hand acupuncture; LF, low frequency; LF/HF, low frequency/high frequency ratio; LF-norm, normalized low frequency LF (LF/LF + HF); n/d = no data; PNS index, parasympathetic nervous system index; PSI, physical stress index; PSS, Perceived Stress Scale; SAS, Self-rating Anxiety Scale; SCL-90R-K-A/D, Symptom Checklist–90–Revision, Korean version; A = anxiety, D = depression; SDRR, standard deviation of RR intervals; SDS, self-rating depression scale; SNS index, sympathetic nervous system index; taVNS, transcutaneous auricular vagus nerve stimulation; VAS, Visual Analogue Scale. ^a^ The effect sizes were calculated based on the mean differences between the groups in the post-test. d = Cohen’s d effect size (When the effect sizes were not available, they have been calculated from means and standard deviations.). *** = *p* < 0.001.

### 3.4. Evaluation of the Quality of Tests

Table 5 and Table 6 provide an assessment of the quality of the studies. The average assessment of the quality of quasi-experimental studies was 6 points (on a scale of 9 points), and for the randomized controlled studies, it was 8 points (on a scale of 13 points). The higher risk of bias in the studies was influenced by unclear information regarding the control of other potential therapeutic interactions (treatment; other therapies; interventions in place). In addition, the studies differed in the methods and the length of HRV measurement and the control of variables that affect HRV values. Therefore, the reliability of HRV measurements increases the risk of error.

## 4. Discussion

The present review identified 12 experimental studies that measured HRV before and after therapeutic intervention. Finally, six studies were analyzed in this review.

The review indicated that taVNS [51], ear acupressure [50], CBT with relaxation elements [46], and yoga (a combination of positions, meditation, and deep diaphragmatic breathing) [48] have an impact on improving the performance of the ANS and reducing the symptoms of IBS (physical and mental). These results are consistent with the evidence from the literature on the effectiveness of individual methods in reducing IBS symptoms.

Diaphragmatic breathing [47] and hand acupuncture [49] did not show the expected effect on autonomic tension and the severity of IBS symptoms. The Jurek study [47] was a pilot study. Subsequent studies are needed to assess the effectiveness of diaphragmatic breathing in IBS therapy. On the other hand, the specificity of the intervention in the Park and Cha study [49] may have made it difficult to obtain a treatment effect (lack of daily stimulation).

If the respiratory rate is described in the characteristics of the intervention, indirectly, the effectiveness of diaphragmatic breathing can be reasoned out from the results of studies on HRV Biofeedback [53,54] or yoga [55]. In biofeedback therapy, it was observed that breathing at a frequency of 0.1 Hz (six breaths per minute) causes a large increase in heart rhythm variability (an increase in the amplitude of heart rate oscillation occurring in the cardiovascular system resulting from the resonance properties of the cardiovascular system) [56], which has a positive impact on the treatment of various clinical groups [57,58]. The study by Jurek et al. [47] confirms the feasibility of slow diaphragmatic breathing training in people with IBS; however, the study did not indicate the frequency of diaphragmatic breathing during training, which limits the conclusions about the effectiveness of the method. Conducting further RCTs (in which the diaphragmatic breathing frequency would be controlled) is advisable, especially since convergence in the mechanisms of the impact of taVNS and diaphragmatic breathing on the vagus nerve is observed. The concurrent use of both interventions may result in additive or synergistic therapeutic effects [59].

Transcutaneous auricular vagus nerve stimulation (taVNS) is a relatively new intervention, the use of which has been successfully tested in the treatment of depression [60] or functional dyspepsia [61]. In a pilot study on the use of taVNS in IBS, a significant reduction in IBS severity (IBS-SS) and pain was observed compared to baseline measurements. The study did not analyze changes in autonomic tone [62]. The measurement of changes in autonomic regulation showed an increase in the vagus nerve efferent activity in patients with functional dyspepsia (HF increase), which coincided with a reduction in the severity of dyspepsia symptoms [61]. The similarity of the taVNS analgesic mechanism and ear acupressure should be noted [63]. The study by Wolf et al. [64] did not confirm the validity of the use of autonomic tone measurements as a biomarker of the effectiveness of taVNS. However, this meta-analysis compared the impact of the intervention on healthy people [64]. This does not exclude the need to further verify the use of this biomarker to measure the effectiveness of interventions used in clinical groups characterized by a reduction in autonomic tone as compared to healthy people [29].

During the review of databases, no research was identified on the effectiveness of other interventions (having a possible impact on autonomic regulation), such as mindfulness [65]; HRV biofeedback [58], hypnosis [66], autogenic training [67], progressive relaxation training [68], and those which would take into account the autonomic measurements. By integrating the results of experimental studies in the scope of MBT’s impact on IBS symptoms, subsequent meta-analyses show that body–mind therapies have the potential to reduce IBS symptoms. However, this evidence is based on self-describing data—the severity of IBS symptoms and/or quality of life and other co-occurring symptoms [16,69].

### 4.1. Characteristics of IBS Patients under Non-Pharmacological Interventions

As shown in Table 4, four studies reported an increase in the activity of the vagus nerve (increased activity of the PNS), as expressed by the HF-HRV index. For example, taVNS increased the vagal activity (HF; normalized values) compared with the sham-taVNS (0.46 ± 0.19 versus 0.34 ± 0.17) [51]. In addition, a decrease in the LF/HF ratio was observed in three studies (out of the five in which it was measured), and a decrease in the LF-HRV ratio was observed in one study (out of four in which it was measured).

The LF to HF ratio was originally tested for the possibility of assessing the ratio of SNS and PNS activity (sympatho-vagal balance) during 24-h HRV measurements. However, the interpretation of this factor is controversial. Moreover, the LF power source is complex (SNS, PNS, and other undefined factors), which makes interpretation difficult [25,70]. Therefore, this review does not interpret the changes in the LF-power and LF/HF ratio. The interpretations were based on the HF-HRV index.

The increase in HF value indicates that the analyzed interventions affect the main pathomechanism (described in Figure 1). In particular, taVNS [51], ear acupressure [50], CBT with relaxation elements [46], and yoga [48] increase parasympathetic activity.

With the increase in vagus nerve activity, there was a decrease in the severity of IBS symptoms. For example, taVNS reduced the IBS-SSS compared with the sham-taVNS (289.5 ± 94.4 versus 197.1 ± 39.6) [51]. Abdominal pain severity decreased (in two out of three studies where it was measured), e.g., taVNS reduced the VAS pain score compared with the sham-taVNS (3.1 ± 2.2 versus 1.1 ± 1.1) [51]. Increased vagal activity in gut–brain–microbiota disorders improved homeostasis by increasing the anti-inflammatory activity of the vagus nerve [11]. As a result, the severity of abdominal pain was reduced [12].

The results of the present review indicated that the intervention increased parasympathetic activity, which means activation of the “rest and digest system” [71]. In addition, changes in HRV induced by effects on the vagus nerve were thought to be an indicator of the body’s ability to regulate and use the adaptive regulation of emotions [27,72]. Beneficial changes in the mental disposition of people with IBS were observed under the influence of the analyzed interventions. People experienced less anxiety (e.g., taVNS reduced the anxiety level (SAS) compared with the sham-taVNS (47.9 ± 9.0 versus 38.7 ± 5.6)) [51], a less depressive mood (e.g., taVNS reduced the depression level (SDS) compared with the sham-taVNS (50.7 ± 11.1 versus 42.6 ± 8.1)) [51], and less stress (e.g., ear-acupressure reduced the stress level (PSS) compared with the non-treatment condition (15.0 ± 3.6 versus 18.3 ± 2.2)) [50].

Assessments of personality and emotional behavior in IBS indicated that anxiety and depression were associated with greater visceral and pain hypersensitivity [73]. It has been shown that a higher level of inflammation (which exacerbates pain experiences) is characteristic of IBS patients with anxiety and depression compared to IBS patients without negative emotions and healthy subjects [74,75]. The changes in physical and mental characteristics observed in this review among IBS patients who underwent complementary medicine interventions fit into the biopsychosocial model of the disorder, and support the need to seek holistic treatment methods.

### 4.2. Heart Rate Variability Measurements

The conducted literature analysis found that the measurement of HRV can be used as a measure of the effectiveness of the therapy, the purpose of which is to influence the efficiency of the ANS. Two types of evidence indicate this. First, the magnitude of the effects of changes between experimental and control groups in post-tests, and their co-occurrence in the scope of changes in: the severity of symptoms of IBS, HRV, and psychological variables (i.e., anxiety, depression, and stress). Second, significant correlations were observed regarding: an increase in parasympathetic activity, along with a decrease in the severity of IBS symptoms (severity of symptoms, pain, anxiety, depression, and stress).

However, the results of the present review should be interpreted carefully due to the diversity in the HRV measurement methodology and possible deviations from the measurement standards. Different HRV measurement methodologies were used in the studies. Changes in resting HRV, as measured by spontaneous respiration, were mostly evaluated. Although the standard short-term measure of HRV is a five-minute measurement [24], other recording lengths were also used in the studies (Table 3), which makes it difficult to directly compare the results.

The research did not focus on the measurement of vagal tone, but on changes in the tone of the ANS in general. Among the measures of autonomic tone, changes in HF were assessed in all studies under the influence of intervention. However, no one reported on the results of the time domain such as RMMSD or pNN50 [26].

In the study by Kavuri, Selvan, Malamud, et al. [48], the HRV measurements were taken during controlled breathing with a constant frequency of six breaths/min. These measurements were aimed at assessing the reactivity of the parasympathetic nervous system; however, the choice of this method was not justified. The HF index, as a measure of vagal tone (including parasympathetic activity), is sensitive to changes in respiratory rate. Malik et al. [24] indicated that HF reflects the vagus nerve tone when the respiratory rate is between nine and 24 breaths per minute (0.15–0.40 Hz). When the respiratory rate exceeds these ranges, it is recommended to assess the RMSSD parameter, which is less sensitive to changes in respiratory rate [76]. Based on the assessment of changes in HRV with the HF index, it is recommended to control the respiratory frequency in the subjects. In the case of the need to change the breathing rate, it is recommended to use the same breathing rate for both compared groups [26].

The studies reported data in different units of measurement of the HRV, which makes it difficult to directly compare the results. As reported in the study, Shaffer and Ginsberg [25], HF can be measured in three units: HF peak—Hz (hertz); HF power ms^2^ (milliseconds squared), or nu (normal/normalized units). The lack of homogeneity in data reporting (or supplements with the results of other parameters) prevents quantitative comparisons of the effectiveness of interventions. In further studies, it is recommended to include detailed data from HRV measurements.

Conducting a reliable HRV measurement requires researchers to check several variables that may affect the results. A detailed list of variables relevant for control during HRV measurements is provided by Laborde et al. [26]. From the perspective of patients with IBS, it is particularly important to control the use of antidepressants due to the significant co-occurrence of depression and IBS. In addition, antidepressants are used for the symptomatic treatment of IBS [77,78,79]. Due to the increased prevalence of IBS among women, it is also advisable to control the use of oral contraceptives and the menstrual cycle phase [80,81]. Due to the sensitivity of short-term HRV measurements to the effects of the daily cycle of the body, it is recommended to perform measurements at the same time of the day (and this applies to both inter- and intra-individual measurements).

To assess the body’s ability to adapt to difficult situations, it is recommended to assess the reactivity of HRV (HRV reactivity) [82]. This methodology was not used by any of the authors of the studies. However, it has already been used as part of the assessment of the effectiveness of IBS therapy [45]. The assessment of changes in HRV reactivity under the influence of therapeutic interventions is interesting from the point of view of assessing changes in the adaptability to environmental challenges at the physiological level. An evaluation of HRV reactivity and resting HRV in the same study may bring additional values to the interpretation of the observed phenomena. The assessment of HRV reactivity is more complicated due to the lack of a homogeneous interpretative framework. Depending on the stressor or the situation in relation to which HRV reactivity is measured, it should be determined whether the decrease or increase in vagal tone will be beneficial for the individual [82].

### 4.3. Limitations of the Review

A small number of studies were analyzed in the review. The review is qualitative, although it is possible to conduct quantitative comparisons of the HRV [29]. However, the studies included in the present review had incomplete data from HRV measurements: different HRV indicators were used and reported in other units and forms of transformations (standard, normalized, logarithmic units). In the present study, we did not provide the results of the within-group changes. Most of the studies were carried out in a scheme of intergroup comparisons. Four studies also reported analyses for within-group changes [48,49,50,51]. When designing future experimental studies and reviews, it is advisable to analyze within-group changes recommended in HRV measurements due to the inter-individual variance of the respondents [26]. When examining the differences in changes in resting HRV before and after therapeutic intervention, it is recommended to calculate individual differences between post-test and pretest measurements, and then compare the means of differences between the study groups, which was performed in the study [50].

The analyzed data refer mainly to the population of women—including young women. Due to the young age of the respondents—the effectiveness of changes in HRV and changes in the severity of IBS symptoms may be unreliable for older age groups. Young age often means a shorter duration of the disease, which results in a shorter time of perpetuation of autonomic changes associated with IBS. This may predispose young people to experience greater benefits in the interventions studied.

### 4.4. Strengths of the Review

The strength of the study is the comparison of the effectiveness of interventions aimed at affecting ANS. Regardless of the therapy used, if it affects ANS, it may benefit patients. The effectiveness of the analyzed research is supported by the currently postulated IBS pathomechanism, i.e., the deregulation of ANS.

Most experimental studies investigate the impact of interventions on the severity of IBS symptoms. The studies analyzed in this review simultaneously observed the impact of the intervention on changes in autonomic tone and the severity of IBS symptoms. Only two studies investigated the interdependence of these changes. However, in both cases, they indicate expected changes. So far, it has not been investigated how ANS mediates the effect of interventions on IBS symptoms. The results of the present review indicated that a mediation analysis can be successfully constructed to more accurately describe the role of therapeutic effects on the ANS in IBS therapy.

### 4.5. Similarities of Analyzed Interventions

Summing up the results of this review, it is worth noting that interventions in which an improvement in the activity of ANS (increase in parasympathetic activity) was observed with a simultaneous reduction of IBS symptoms had several common features:

(a) stimulation of the vagus nerve, which is recommended in IBS therapy [10] (the CBT therapy with relaxation elements has the potential to affect the vagal tone as well);
(b) the starting point of the observed changes was the stimulation of peripheral sensory nerve fibers in the skin, muscles, and viscera (bottom-up process). Taylor et al. [83] postulate that bottom-up processes in MBT interventions correct functional changes in central nervous processing, increase heart rate variability (HRV), and decrease the expression of proinflammatory cytokines;
(c) they took place daily or enabled the daily implementation of acquired skills.

## 5. Conclusions

The results of the review indicate that it is necessary to further analyze the possibilities of IBS therapies, with particular emphasis on therapies stimulating the vagus nerve. It is advisable to plan experimental studies with the perspective of their subsequent synthesis in the form of meta-analysis. For this purpose, it will be helpful to standardize HRV measures reported in the test results and/or to attach measurement results in the form of databases and supplements to published articles. This will not only help to obtain data on HRV as a measure of the effectiveness of therapies, but also develop evidence-based interventions that affect ANS.

The next step in the research is to identify the individual characteristics of people who are more susceptible to individual forms of therapy. Research on the assessment of predispositions—including autonomic ones—to achieve benefits in MBT therapies has already begun [34,84]. Performing HRV measurements as a flagship indicator of the effectiveness of IBS therapy will allow determining, in the future, which people can benefit most from therapies aiming at ANS. As a result, it will improve the process of selecting effective forms of IBS treatment, and, thus, reduce the psychosocial and economic costs generated by IBS.

## Figures and Tables

**Table 1:** General characteristics of research studies included in the review.

Author	Year	Country	N (E/C)	% Women	Rome Criteria	IBS Type	Methods for Assessing IBS Symptoms	Methods for Assessing Secondary Outcomes
Jang et al. [46]	2017	Korea	21/17	100	III	IBS-C	GI Symptom Rating Scale (GSRS-IBS)	Anxiety, Depression: HADS; Stress: GARS
Jurek et al. [47]	2021	USA	7/6	69.2	n/d	n/d	IBS Severity Scoring System (IBS-SSS)	none
Kavuri, Selvan, Malamud et al. [48]	2015	USA	25/26/27	83.3	III	IBS-C/D/M	IBS Severity Scoring System (IBS-SSS)	Anxiety, Depression: HADS
Park and Cha [49]	2012	Korea	21/21	100	III	n/d	IBS module from Rome III Questionnaire (10 items); Bowel Symptom Severity Scale (BSSS)	Stress: GARS; Mental Health: SCL-90R-K
Go and Park [50]	2019	South Korea	29/27	100	III	IBS-C/D/M	Bowel Symptom Severity Scale (BSSS)	Stress: PSS; Mental Health: SCL-90R-K
Shi et al. [51]	2021	China	21/19	75	IV	IBS-C	IBS-SSS, Bristol stool form scale (BSFS), the bowel diary with Visual Analogue Scale (VAS) for abdominal pain	Anxiety/Depression: SAS/SDS

C = control group; E = experimental group; GARS = Global Assessment of Recent Stress; HADS = Hospital Anxiety and Depression Scale; IBS-C = IBS with predominant constipation; IBS-D = IBS with predominant diarrhea; IBS-M = IBS with mixed bowel habits; n/d = no data; PSS = Perceived Stress Scale; SAS/SDS = the ZungSelf-rating Anxiety and Depression Scale; SCL-90R-K = Symptom Checklist–90–Revision, Korean version.

**Table 5:** Results of JBI Critical Appraisal for Quasi-Experimental Studies.

Study	1	2	3	4	5	6	7	8	9	Overall ^a^
Park & Cha [49]	+	+	?	+	+	−	+	?	+	6.00
Go & Park [50]	+	+	+	+	+	?	+	?	+	7.00
Jurek et al. [47]	+	−	?	+	+	?	+	?	+	5.00

*Note.* +, criterion fulfilled; −, criterion not fulfilled; ?, unclear; ^a^ Interpretation: 0—high; 9—low risk of bias.

**Table 6:** Results of JBI Critical Appraisal for Randomized Controlled Trials.

Study	1	2	3	4	5	6	7	8	9	10	11	12	13	Overall ^a^
Jang et al. [46]	+	?	+	?	-	+	−	?	−	+	?	+	+	6.00
Kavuri, Selvan, Malamud et al. [48]	+	+	+	?	+	+	−	?	+	+	?	+	+	9.00
Shi et al. [51]	+	−	+	+	−	−	+	+	+	+	?	+	+	9.00

*Note.* +, criterion fulfilled; −, criterion not fulfilled; ?, unclear; ^a^ Interpretation: 0—high; 13—low risk of bias.

## Data Availability

The dataset used during the current study are available from the corresponding author on reasonable request.
